# Role of Host MicroRNAs in Kaposi’s Sarcoma-Associated Herpesvirus Pathogenesis

**DOI:** 10.3390/v6114571

**Published:** 2014-11-21

**Authors:** Zhiqiang Qin, Francesca Peruzzi, Krzysztof Reiss, Lu Dai

**Affiliations:** 1 Research Center for Translational Medicine and Key Laboratory of Arrhythmias of the Ministry of Education of China, East Hospital, Tongji University School of Medicine, 150 Jimo Road, Shanghai 200120, China; 2 Department of Microbiology, Immunology and Parasitology, Louisiana State University Health Sciences Center, Louisiana Cancer Research Center, 1700 Tulane Ave., New Orleans, LA 70112, USA; 3 Neurological Cancer Research, Stanley S. Scott Cancer Center, Department of Medicine, Louisiana State University Health Sciences Center, 1700 Tulane Ave., New Orleans, LA 70112, USA; E-Mails: fperuz@lsuhsc.edu (F.P.); kreiss@lsuhsc.edu (K.R.)

**Keywords:** KSHV, microRNA, pathogenesis

## Abstract

MicroRNAs (miRNAs) are small non-coding RNA species that can bind to both untranslated and coding regions of target mRNAs, causing their degradation or post-transcriptional modification. Currently, over 2500 miRNAs have been identified in the human genome. Burgeoning evidence suggests that dysregulation of human miRNAs can play a role in the pathogenesis of a variety of diseases, including cancer. In contrast, only a small subset of human miRNAs has been functionally validated in the pathogenesis of oncogenic viruses, in particular, Kaposi’s sarcoma-associated herpesvirus (KSHV). KSHV is the etiologic agent of several human cancers, such as primary effusion lymphoma (PEL) and Kaposi’s sarcoma (KS), which are mostly seen in acquired immune deficiency syndrome (AIDS) patients or other immuno-suppressed subpopulation. This review summarizes recent literature outlining mechanisms for KSHV/viral proteins regulation of cellular miRNAs contributing to viral pathogenesis, as well as recent findings about the unique signature of miRNAs induced by KSHV infection or KSHV-related malignancies.

## 1. Introduction

MicroRNAs (miRNAs) are small (~19–24 nucleotides in length), non-coding RNAs that bind to both, untranslated and coding regions of target mRNAs, causing their degradation or post-transcriptional modification. The biogenesis of miRNAs begins in the nucleus where RNA polymerase II generates primary miRNA (pri-miRNAs) transcripts, which are subsequently processed by the RNase III enzyme Drosha, generating precursor miRNAs (pre-miRNAs). Pre-miRNAs are transported from the nucleus to the cytoplasm where they are cleaved by the cytoplasmic RNase III enzyme, Dicer, generating mature miRNAs, which are incorporated into the RNA-induced silencing complex (RISC) [1,2]. Published literature has demonstrated that miRNAs regulate a variety of physiological and pathological processes in the cell, such as cell proliferation, apoptosis, differentiation, development, mobility, invasiveness, and angiogenesis [3].

miRNAs are encoded by many different organisms including viruses, in which miRNA sequences and functions are often different from human miRNAs [4]. For example, several oncogenic herpesviruses have been found encoding multiple miRNAs in their genomes, such as Kaposi’s sarcoma-associated herpesvirus (KSHV), which is the etiologic agent of human cancers including multicentric Castleman’s disease (MCD), primary effusion lymphoma (PEL), and Kaposi’s sarcoma (KS) [5,6,7]. Thus far, 12 KSHV pre-miRNAs (miR-K12-1~miR-K12-12), encoding 18 mature miRNAs, have been identified within the viral genome [8,9]. These miRNAs are located in the KSHV latency-associated region (KLAR), together with several KSHV-encoded latent proteins, which are critical for maintenance of the viral episome and for KSHV-mediated oncogenesis. In fact, most of these KSHV-miRNAs are expressed in different KSHV-infected host cells and/or KSHV-related tumor tissues, and play important roles in viral pathogenesis and tumorigenesis, which have been comprehensively reviewed by us and others before [10,11,12,13]. Interestingly, several KSHV-miRNAs, including miR-K12-11, miR-K12-10a and miR-K12-3, can act as viral analogs of the human cellular miRNAs miR-155, miR-142-3p and miR-23, respectively. As a result of such homology in the seeding sequence, cellular and viral miRNAs can share the repertoire of targeted genes [14,15,16,17]. In contrast to the well-defined KSHV-miRNAs, we know little about how human miRNAs can be regulated by KSHV/viral proteins and functionally involved in viral pathogenesis and tumorigenesis. The current review will summarize recent findings regarding the regulatory function of human miRNAs contributing to the pathogenesis of KSHV-infected host cells and KSHV-related malignancies.

## 2. Human miRNAs and KSHV-Induced Cell Mobility and Angiogenesis

Acquisition of a migratory or invasive phenotype represents one of the hallmarks of KSHV-infected endothelial cells, with implications for both, viral dissemination and angiogenesis within KS lesions [18,19]. Moreover, KS is characterized by the proliferation of infected spindle cells of vascular and lymphatic endothelial origin, accompanied by intense angiogenesis with erythrocyte extravasation and inflammatory infiltration [20,21]. Recent reports provide the solid evidence that cellular miRNAs are involved in KSHV-induced cell mobility and angiogenesis. Tsai and colleagues reported that KSHV-encoded K15 protein, minor form (K15M), can induce cell migration and invasion, potentially through upregulation of cellular miR-21 and miR-31 via its conserved Src-Homology 2 (SH2)-binding motif [22]. In contrast, knocking down both miR-21 and miR-31 inhibited K15M-mediated cell motility, which indicated that targeting K15 or its downstream-regulated microRNAs may represent novel therapies for treatment of KSHV-associated neoplasia [22]. Upregulation of miR-31 by KSHV was further confirmed in virally infected lymphatic endothelial cells (LECs), in which depletion of miR-31 reduced cell mobility [23]. One of the mechanisms identified for the miR-31 mediated increasing cell motility was through direct repression of a novel tumor suppressor and inhibitor of migration, FAT4; moreover, a reduction of FAT4 enhanced EC mobility [23]. Through the analysis of miRNAs microarray data, the miR-221/miR-222 cluster was found significantly downregulated in KSHV-infected LECs and resulted in an increase of EC migration, potentially through KSHV-encoded latency-associated nuclear antigen (LANA) and Kaposin B proteins [23]. Further experimental data confirmed that the transcription factors, ETS2 and ETS1, were the downstream targets of miR-221 and miR-222, respectively, and overexpression of ETS1 or ETS2 alone was sufficient to induce EC migration [23]. In addition to these factors, KSHV can also downregulate miR-30b and miR-30c, whereas increasing the expression of their direct target, Delta-like 4 (DLL4), a functional protein in vascular development and angiogenesis [24], can induce KSHV-mediated LECs angiogenesis [25]. Interestingly, these miRNAs (miR-21, miR-31, miR-221/222, miR-30) can act as either “oncogenes” or “tumor-suppressor genes” in a variety of cancers in which they can regulate tumor cell proliferation, apoptosis, invasion, angiogenesis, metastasis and other important cellular functions [26,27,28,29,30], indicating functional relevance of these regulatory miRNAs in virus-related malignancies.

## 3. Human miRNAs and KSHV Lifecycle/Replication

KSHV lifecycle involves two distinct phases: latent and lytic. During latent infection, which represents the predominant phase in the majority of infected cells, only a limited number of viral genes are expressed. Exposure to a variety of stimuli induces lytic replication, resulting in virion assembly and release of infectious viral particles [31]. Maintenance of latent KSHV infection, coordinated with lytic reactivation within a small subset of infected cells, is critical for promotion of KSHV persistence and dissemination. Several published studies have demonstrated a role for specific KSHV-miRNAs in maintaining viral latency through either direct targeting of the viral lytic reactivation activator, *Rta* (ORF50) [32,33], or via indirect mechanisms including targeting host factors such as IκBα, nuclear factor I/B (NFIB), Rbl2, BCLAF1, and IKKɛ [34,35,36,37,38]. In contrast, little is known about the role of human miRNAs regulating “latent to lytic” switch in KSHV lifecycle. Recent miRNA profiling studies indicated that ectopic expression of HIV-encoded Nef protein can suppress the expression of KSHV lytic proteins and the production of infectious viral particles, potentially through regulation of cellular miRNAs [39]. Indeed, at least five of the 99 miRNAs upregulated by Nef (miR-557, miR-766, miR-1227, miR-1258, and miR-1301) had putative binding sites in the 3’ UTR of viral lytic reactivation activator, *Rta* [39]. Further data confirmed that ectopic expression of miR-1258 impaired RTA synthesis and enhanced Nef-mediated inhibition of KSHV replication [39]. Based on the complex mechanisms (direct and indirect) for KSHV lytic reactivation mentioned above, it is reasonable to conceive that there should be even more cellular miRNAs involved in the regulation of KSHV lifecycle and replication.

## 4. Human miRNAs and KSHV-Induced Cytokine Response and Immune Recognition

KSHV infection can induce a variety of pro-migratory, pro-angiogenic and pro-inflammatory cytokines and chemokines to promote viral pathogenesis and survival of the infected cells [21]. Moreover, to establish a life-long persistent infection, KSHV has evolved a complex mechanism by which unique viral proteins antagonize host innate and adaptive immunity [40]. For example, KHSV infection and ectopic expression of KSHV-encoded viral FLICE inhibitory protein (vFLIP) suppressed the expression of one chemokine receptor, CXCR4 [41]. Suppression of CXCR4 by KSHV and vFLIP was associated with the upregulation of cellular miR-146a expression, a miRNA that is known to bind to the 3’UTR of CXCR4 mRNA. Further data confirmed that upregulation of miR-146a required vFLIP-induced NF-κB activities, because vFLIP NF-κB-defective mutant lost such ability. For clinical relevance, downregulation of CXCR4 accompanied by increased expression of miR-146a has been found in the KS tissues derived from patients, and it could contribute to KS development by promoting premature release of KSHV-infected endothelial progenitors into the circulation [41].

KSHV encodes a viral interleukin 6 (vIL-6) that mimics many functions of human IL-6 (hIL-6), since they can both stimulate the proliferation of tumors caused by KSHV and play a role in the inflammatory cytokine syndrome associated with HIV and KSHV co-infection [42,43,44,45]. Kang and colleagues recently identified a direct repression of vIL6 by cellular miR-1293 and hIL6 by miR-608 through binding sites in their ORF sequences [46]. More importantly, miR-1293 is primarily expressed in the germinal center, but is not present in the mantle zone of human lymph nodes where the expression of vIL6 is often found in patients with KSHV-associated MCD [46]. Interestingly, the KSHV-encoded ORF57 protein appeared to compete with miR-1293 and/or miR-608 for the same binding site in the vIL-6 and/or hIL-6 RNAs, thereby preventing vIL-6 and/or hIL-6 RNA degradation from association with the miR-1293/miR-608-specified RISC [47]. These data have demonstrated that KSHV has evolved a “smart strategy” by using viral proteins against host miRNAs regulation to its own advantage, including mechanisms that would allow survival of infected cells and promote virus-associated malignancies.

In addition, Lagos *et al.* reported two groups of cellular miRNAs induced during primary KSHV infection of LECs: the “early” group reached its peak of expression at six hours post-infection, and included miR-146a, miR-31 and miR-132; the “late” group, which included miR-193a and Let-7i, steadily increased its expression during the next 72 hours [48]. One of the lately expressed miRNAs, the highly upregulated miR-132, has been shown to negatively affect the expression of interferon-stimulated genes through suppression of the p300 transcriptional co-activator, facilitating viral gene expression and replication [48]. These data clearly indicate that this oncogenic virus can use host miRNAs to regulate antiviral innate immunity to promote survival of the infected cells. Interestingly, a similar induction of functional miR-132 was also observed during infection of monocytes with herpes simplex virus-1 (HSV-1) and cytomegalovirus (CMV) [48], which indicated that this kind of miRNA-mediated antiviral immune response could be triggered by several viruses.

## 5. Human miRNAs Profile in KSHV-Related Malignancies

miRNA microarray combined with bioinformatics analysis represents a powerful tool to understand miRNA global profile unique to certain cancer cells/tissues when compared with normal controls. Recent studies using different miRNA microarray have gained insight into the cellular miRNAs profile altered in KSHV-related malignancies including KS and PEL. Wu *et al.* performed a miRNA profiling by analyzing six paired KS and matched adjacent healthy tissues using the miRCURY^TM^ LNA Array (v.18.0) (Exiqon, Woburn, MA, USA) which contains 3100 capture probes, covering all human, mouse, and rat miRNAs annotated in miRBase 18.0, as well as all viral microRNAs related to these species [49]. They identified 170 differentially expressed miRNAs (69 upregulated and 101 downregulated) in KS *versus* adjacent healthy tissues. Among them, the most significantly upregulated human miRNAs included miR-126-3p, miR-199a-3p, and miR-16-5p, while the most significantly downregulated miRNAs included miR-125b-1-3p and miR-1183. Of those, miR-125b-1-3p and miR-16-5p had statistically significant associations with KSHV and HIV infections in KS. Catrina Ene and colleagues performed miRNA microarray analysis of 17 KS specimens and three normal skin specimens as controls, using the miRNA Microarray Kit V2 platform which contains 723 human and 76 human viral miRNAs from the Sanger database v.10.1 (Agilent Technologies, Santa Clara, CA, USA) [50]. They detected 185 differentially expressed miRNAs in KS *versus* normal skin; of those, 76 were upregulated and 109 were downregulated. The most significantly upregulated human miRNAs were miR-513a-3p, miR-298, and miR-206; whereas miR-99a, miR-200 family, miR-199b-5p, miR-100, and miR-335 were the most significantly downregulated miRNAs. We assume that the differential signature of miRNAs found in KS samples from these two studies is probably caused by the different patient race, geography area (the first study was conducted in Asia and the second was in Europe), miRNA microarray platforms and data analysis methods. Although informative, perhaps larger and more systematic studies should be designed to find a definitive miRNA signature that could distinguish those different KS subtypes, including classic KS, endemic KS, iatrogenic KS, and epidemic KS (AIDS-KS) [21].

In another KSHV-caused malignancy, PEL, O’Hara *et al.* identified 68 PEL-specific miRNAs by using a TaqMan-based miRNA array [51]. Interestingly, some tumor suppressor miRNAs, including miR-221/222 and let-7 family members, were found significantly downregulated in KSHV-related malignancies, such as PEL and KS [52]. Therefore, downregulation of these tumor suppressor miRNAs may represent an alternative mechanism of KSHV-mediated transformation.

In addition to intracellular miRNAs, circulating miRNAs such as those found in exosomes, have emerged as powerful diagnostic tools and may act as minimally invasive, stable biomarkers. Transfer of tumor-derived exosomal miRNAs to surrounding cells may represent an important form of cellular communication [53,54,55]. Recently, Chugh and colleagues measured the host circulating miRNAs in plasma, pleural fluid or serum from patients with KS or PEL and from two mouse models of KS [56]. They found that many host miRNAs in particular the members of miR-17-92 cluster, were detectable within patient exosomes and circulating miRNA profiles from KSHV mouse models. Moreover, a subset of miRNAs including miR-19a, miR-21, miR-27a, miR-130, and miR-146a, seemed to be preferentially incorporated into exosomes, suggesting their potential use as biomarkers for KSHV-associated diseases [56]. In addition to circulating miRNAs and exosomes, more than 100 cellular miRNAs and abundant U2 small nuclear RNAs (snRNA)-derived unusual small RNAs (usRNAs) were detected in KSHV virions using the deep-sequencing technology [57]. Similarly, some miRNAs including miR-143, miR-23a, miR-130b, miR-451, and miR-185, were found preferentially packaged into KSHV virions when compared with intracellular miRNAs profile in KSHV-infected cells [57]. Taken together, these recent findings indicate that KSHV-related malignancies have a unique signature, not only for intracellular miRNAs, but also extracellular miRNAs (circulating and virion-packed miRNAs), although the underlying mechanisms and individual miRNA-mediated functions remain largely unknown within these malignancies.

## 6. Concluding Remarks

Until now, over 2500 human miRNAs have been identified and the list is still growing based on the miRBase database [58]. However, only a very small subset of human miRNAs as “tip of the iceberg” has been functionally validated in KSHV-infected cells or KSHV-related malignancies (summarized in Table 1). In fact, recent array-based data have indicated that either KSHV infection or KSHV-related malignancies can induce the unique signature of human intracellular and extracellular miRNAs [49,50,51,52,56,57], suggesting that more host miRNAs are likely involved in the regulation of KSHV pathogenesis. Functional validation of these human miRNAs and their respective target genes in future investigations will eventually help to better understand this virus-host interaction network and provide the framework for the development of more effective strategies targeting host miRNAs-mediated regulatory network against KSHV-related diseases.

**Table 1:** Overview of human miRNAs regulated by KSHV/viral proteins.

Human miRNAs	Validated Targets	Regulated by Viral Proteins	Functions	References
miR-21	-	K15M	Cell mobility	[22]
miR-31	FAT4	K15M	Cell mobility	[22,23]
miR-221/222	ETS2/ETS1	LANA and Kaposin B	Cell migration	[23]
miR-30b/c	DLL4	-	Angiogenesis	[25]
miR-557/766/1227/1258/1301	RTA	-	Viral replication	[39]
miR-146a	CXCR4	vFLIP	Immune response	[41]
miR-1293	vIL-6	ORF57	Immune response	[46,47]
miR-608	hIL-6	ORF57	Immune response	[46,47]
miR-132	p300	-	Immune escape	[48]

## References

[1] Moens U. (2009). Silencing viral microRNA as a novel antiviral therapy?. J. Biomed. Biotechnol..

[2] Bartel D.P. (2004). MicroRNAs: Genomics, biogenesis, mechanism, and function. Cell.

[3] Ambros V. (2004). The functions of animal microRNAs. Nature.

[4] Cullen B.R. (2009). Viral and cellular messenger RNA targets of viral microRNAs. Nature.

[5] Soulier J., Grollet L., Oksenhendler E., Cacoub P., Cazals-Hatem D., Babinet P., d’Agay M.F., Clauvel J.P., Raphael M., Degos L. (1995). Kaposi’s sarcoma-associated herpesvirus-like DNA sequences in multicentric Castleman’s disease. Blood.

[6] Cesarman E., Chang Y., Moore P.S., Said J.W., Knowles D.M. (1995). Kaposi’s sarcoma-associated herpesvirus-like DNA sequences in AIDS-related body-cavity-based lymphomas. N. Engl. J. Med..

[7] Chang Y., Cesarman E., Pessin M.S., Lee F., Culpepper J., Knowles D.M., Moore P.S. (1994). Identification of herpesvirus-like DNA sequences in AIDS-associated Kaposi’s sarcoma. Science.

[8] Cai X., Lu S., Zhang Z., Gonzalez C.M., Damania B., Cullen B.R. (2005). Kaposi’s sarcoma-associated herpesvirus expresses an array of viral microRNAs in latently infected cells. Proc. Natl. Acad. Sci. USA.

[9] Samols M.A., Hu J., Skalsky R.L., Renne R. (2005). Cloning and identification of a microRNA cluster within the latency-associated region of Kaposi’s sarcoma-associated herpesvirus. J. Virol..

[10] Zhu Y., Haecker I., Yang Y., Gao S.J., Renne R. (2013). gamma-Herpesvirus-encoded miRNAs and their roles in viral biology and pathogenesis. Curr. Opin. Virol..

[11] Qin Z., Jakymiw A., Findlay V., Parsons C. (2012). KSHV-Encoded MicroRNAs: Lessons for Viral Cancer Pathogenesis and Emerging Concepts. Int. J. Cell Biol..

[12] Ziegelbauer J.M. (2011). Functions of Kaposi’s sarcoma-associated herpesvirus microRNAs. Biochim Biophys Acta.

[13] Ganem D., Ziegelbauer J. (2008). MicroRNAs of Kaposi’s sarcoma-associated herpes virus. Semin. Cancer Biol..

[14] Gottwein E., Corcoran D.L., Mukherjee N., Skalsky R.L., Hafner M., Nusbaum J.D., Shamulailatpam P., Love C.L., Dave S.S., Tuschl T. (2011). Viral microRNA targetome of KSHV-infected primary effusion lymphoma cell lines. Cell Host Microbe.

[15] Gottwein E., Mukherjee N., Sachse C., Frenzel C., Majoros W.H., Chi J.T., Braich R., Manoharan M., Soutschek J., Ohler U. (2007). A viral microRNA functions as an orthologue of cellular miR-155. Nature.

[16] Skalsky R.L., Samols M.A., Plaisance K.B., Boss I.W., Riva A., Lopez M.C., Baker H.V., Renne R. (2007). Kaposi’s sarcoma-associated herpesvirus encodes an ortholog of miR-155. J. Virol..

[17] Manzano M., Shamulailatpam P., Raja A.N., Gottwein E. (2013). Kaposi’s sarcoma-associated herpesvirus encodes a mimic of cellular miR-23. J. Virol..

[18] Qin Z., Dai L., Slomiany M.G., Toole B.P., Parsons C. (2010). Direct activation of emmprin and associated pathogenesis by an oncogenic herpesvirus. Cancer Res..

[19] Qin Z., Dai L., Defee M., Findlay V.J., Watson D.K., Toole B.P., Cameron J., Peruzzi F., Kirkwood K., Parsons C. (2013). Kaposi’s Sarcoma-Associated Herpesvirus Suppression of DUSP1 Facilitates Cellular Pathogenesis following De Novo Infection. J. Virol..

[20] Ganem D. (2010). KSHV and the pathogenesis of Kaposi sarcoma: Listening to human biology and medicine. J. Clin. Investig..

[21] Mesri E.A., Cesarman E., Boshoff C. (2010). Kaposi’s sarcoma and its associated herpesvirus. Nat. Rev. Cancer.

[22] Tsai Y.H., Wu M.F., Wu Y.H., Chang S.J., Lin S.F., Sharp T.V., Wang H.W. (2009). The M type K15 protein of Kaposi’s sarcoma-associated herpesvirus regulates microRNA expression via its SH2-binding motif to induce cell migration and invasion. J. Virol..

[23] Wu Y.H., Hu T.F., Chen Y.C., Tsai Y.N., Tsai Y.H., Cheng C.C., Wang H.W. (2011). The manipulation of miRNA-gene regulatory networks by KSHV induces endothelial cell motility. Blood.

[24] Phng L.K., Potente M., Leslie J.D., Babbage J., Nyqvist D., Lobov I., Ondr J.K., Rao S., Lang R.A., Thurston G. (2009). Nrarp coordinates endothelial Notch and Wnt signaling to control vessel density in angiogenesis. Dev. Cell.

[25] Bridge G., Monteiro R., Henderson S., Emuss V., Lagos D., Georgopoulou D., Patient R., Boshoff C. (2012). The microRNA-30 family targets DLL4 to modulate endothelial cell behavior during angiogenesis. Blood.

[26] Sekar D., Hairul Islam V.I., Thirugnanasambantham K., Saravanan S. (2014). Relevance of miR-21 in HIV and non-HIV-related lymphomas. Tumour Biol..

[27] Laurila E.M., Kallioniemi A. (2013). The diverse role of miR-31 in regulating cancer associated phenotypes. Genes Chromosomes Cancer.

[28] Chen W.X., Hu Q., Qiu M.T., Zhong S.L., Xu J.J., Tang J.H., Zhao J.H. (2013). miR-221/222: Promising biomarkers for breast cancer. Tumour Biol..

[29] Garofalo M., Quintavalle C., Romano G., Croce C.M., Condorelli G. (2012). miR221/222 in cancer: Their role in tumor progression and response to therapy. Curr. Mol. Med..

[30] Fuziwara C.S., Kimura E.T. (2014). MicroRNA Deregulation in Anaplastic Thyroid Cancer Biology. Int. J. Endocrinol..

[31] Schulz T.F. (2006). The pleiotropic effects of Kaposi’s sarcoma herpesvirus. J. Pathol..

[32] Lin X., Liang D., He Z., Deng Q., Robertson E.S., Lan K. (2011). miR-K12–7-5p encoded by Kaposi’s sarcoma-associated herpesvirus stabilizes the latent state by targeting viral ORF50/RTA. PLoS One.

[33] Bellare P., Ganem D. (2009). Regulation of KSHV lytic switch protein expression by a virus-encoded microRNA: An evolutionary adaptation that fine-tunes lytic reactivation. Cell Host Microbe.

[34] Lei X., Bai Z., Ye F., Xie J., Kim C.G., Huang Y., Gao S.J. (2010). Regulation of NF-kappaB inhibitor IkappaBalpha and viral replication by a KSHV microRNA. Nat. Cell. Biol..

[35] Lu C.C., Li Z., Chu C.Y., Feng J., Sun R., Rana T.M. (2010). MicroRNAs encoded by Kaposi’s sarcoma-associated herpesvirus regulate viral life cycle. EMBO Rep..

[36] Lu F., Stedman W., Yousef M., Renne R., Lieberman P.M. (2010). Epigenetic regulation of Kaposi’s sarcoma-associated herpesvirus latency by virus-encoded microRNAs that target Rta and the cellular Rbl2-DNMT pathway. J. Virol..

[37] Liang D., Gao Y., Lin X., He Z., Zhao Q., Deng Q., Lan K. (2011). A human herpesvirus miRNA attenuates interferon signaling and contributes to maintenance of viral latency by targeting IKKepsilon. Cell Res..

[38] Ziegelbauer J.M., Sullivan C.S., Ganem D. (2009). Tandem array-based expression screens identify host mRNA targets of virus-encoded microRNAs. Nat. Genet..

[39] Yan Q., Ma X., Shen C., Cao X., Feng N., Qin D., Zeng Y., Zhu J., Gao S.J., Lu C. (2014). Inhibition of Kaposi’s sarcoma-associated herpesvirus lytic replication by HIV-1 Nef and cellular microRNA hsa-miR-1258. J. Virol..

[40] Lee H.R., Lee S., Chaudhary P.M., Gill P., Jung J.U. (2010). Immune evasion by Kaposi’s sarcoma-associated herpesvirus. Future Microbiol..

[41] Punj V., Matta H., Schamus S., Tamewitz A., Anyang B., Chaudhary P.M. (2010). Kaposi’s sarcoma-associated herpesvirus-encoded viral FLICE inhibitory protein (vFLIP) K13 suppresses CXCR4 expression by upregulating miR-146a. Oncogene.

[42] Aoki Y., Yarchoan R., Wyvill K., Okamoto S., Little R.F., Tosato G. (2001). Detection of viral interleukin-6 in Kaposi sarcoma-associated herpesvirus-linked disorders. Blood.

[43] Jones K.D., Aoki Y., Chang Y., Moore P.S., Yarchoan R., Tosato G. (1999). Involvement of interleukin-10 (IL-10) and viral IL-6 in the spontaneous growth of Kaposi’s sarcoma herpesvirus-associated infected primary effusion lymphoma cells. Blood.

[44] Nicholas J., Ruvolo V.R., Burns W.H., Sandford G., Wan X., Ciufo D., Hendrickson S.B., Guo H.G., Hayward G.S., Reitz M.S. (1997). Kaposi’s sarcoma-associated human herpesvirus-8 encodes homologues of macrophage inflammatory protein-1 and interleukin-6. Nat. Med..

[45] Uldrick T.S., Wang V., O’Mahony D., Aleman K., Wyvill K.M., Marshall V., Steinberg S.M., Pittaluga S., Maric I., Whitby D. (2010). An interleukin-6-related systemic inflammatory syndrome in patients co-infected with Kaposi sarcoma-associated herpesvirus and HIV but without Multicentric Castleman disease. Clin. Infect. Dis..

[46] Kang J.G., Majerciak V., Uldrick T.S., Wang X., Kruhlak M., Yarchoan R., Zheng Z.M. (2011). Kaposi’s sarcoma-associated herpesviral IL-6 and human IL-6 open reading frames contain miRNA binding sites and are subject to cellular miRNA regulation. J. Pathol..

[47] Kang J.G., Pripuzova N., Majerciak V., Kruhlak M., Le S.Y., Zheng Z.M. (2011). Kaposi’s sarcoma-associated herpesvirus ORF57 promotes escape of viral and human interleukin-6 from microRNA-mediated suppression. J. Virol..

[48] Lagos D., Pollara G., Henderson S., Gratrix F., Fabani M., Milne R.S., Gotch F., Boshoff C. (2010). miR-132 regulates antiviral innate immunity through suppression of the p300 transcriptional co-activator. Nat. Cell Biol..

[49] Wu X.J., Pu X.M., Zhao Z.F., Zhao Y.N., Kang X.J., Wu W.D., Zou Y.M., Wu C.Y., Qu Y.Y., Zhang D.Z. (2014). The expression profiles of microRNAs in Kaposi's sarcoma. Tumour Biol..

[50] Catrina Ene A.M., Borze I., Guled M., Costache M., Leen G., Sajin M., Ionica E., Chitu A., Knuutila S. (2014). MicroRNA expression profiles in Kaposi’s sarcoma. Pathol. Oncol. Res..

[51] O’Hara A.J., Vahrson W., Dittmer D.P. (2008). Gene alteration and precursor and mature microRNA transcription changes contribute to the miRNA signature of primary effusion lymphoma. Blood.

[52] O’Hara A.J., Wang L., Dezube B.J., Harrington W.J., Damania B., Dittmer D.P. (2009). Tumor suppressor microRNAs are underrepresented in primary effusion lymphoma and Kaposi sarcoma. Blood.

[53] Moltzahn F., Olshen A.B., Baehner L., Peek A., Fong L., Stoppler H., Simko J., Hilton J.F., Carroll P., Blelloch R. (2011). Microfluidic-based multiplex qRT-PCR identifies diagnostic and prognostic microRNA signatures in the sera of prostate cancer patients. Cancer Res..

[54] Huang Z., Huang D., Ni S., Peng Z., Sheng W., Du X. (2010). Plasma microRNAs are promising novel biomarkers for early detection of colorectal cancer. Int. J. Cancer.

[55] Mitchell P.S., Parkin R.K., Kroh E.M., Fritz B.R., Wyman S.K., Pogosova-Agadjanyan E.L., Peterson A., Noteboom J., O’Briant K.C., Allen A. (2008). Circulating microRNAs as stable blood-based markers for cancer detection. Proc. Natl. Acad. Sci. USA.

[56] Chugh P.E., Sin S.H., Ozgur S., Henry D.H., Menezes P., Griffith J., Eron J.J., Damania B., Dittmer D.P. (2013). Systemically circulating viral and tumor-derived microRNAs in KSHV-associated malignancies. PLoS Pathog..

[57] Lin X., Li X., Liang D., Lan K. (2012). MicroRNAs and unusual small RNAs discovered in Kaposi’s sarcoma-associated herpesvirus virions. J. Virol..

[58] Acunzo M., Romano G., Wernicke D., Croce C.M. (2014). MicroRNA and cancer—A brief overview. Adv. Biol. Regul..

